# Length polymorphism in *OGT* between Korean native pig, Chinese Meishan, and the Western pig breeds

**DOI:** 10.1186/s40781-015-0045-5

**Published:** 2015-03-14

**Authors:** Yoon Seok Nam, Doo-Wan Kim, Myoung-Jik Kim, Kyu-Ho Cho, Jong Gug Kim

**Affiliations:** Department of Animal Sciences, College of Agriculture and Life Science, and Institute of Molecular Biology and Genetics, Chonbuk National University, Jeonju-si, Jeollabuk-do 561-756 Republic of Korea; Swine Science Division, National Institute of Animal Science, RDA, 114 Sinbang 1-gil, Seonghwan-eup, Seobuk-gu, Cheonan-si, Chungnam 331-801 Republic of Korea; 567 Baekje-Daero, Deokjin-gu, Jeonju-si, Jeollabuk-do 561-756 Republic of Korea

**Keywords:** O-linked N-acetylglucosamine transferase, OGT, Length polymorphism, KNP, Genotype

## Abstract

**Background:**

The Korean native pig (KNP) is generally thought to have come from northern China to the Korean peninsula approximately 2000 years ago. KNP pigs were at the brink of extinction in the 1980s, since then efforts have been made to restore the breed by bringing together the remaining stocks in South Korea. As a result, KNP was registered as a breed in 2006. To find additional breed-specific markers that are distinct among pig breeds, variations in *O-linked N-acetylglucosamine transferase* (*OGT*) were investigated. *OGT* is located on chromosome X and catalyzes the post-translational addition of a single O-linked-β-N-acetylglucosamine to target proteins.

**Findings:**

Length polymorphism in the intron 20 of *OGT* was identified. The intron 20 of *OGT* from Duroc, Landrace, and Yorkshire breeds was 281-bp longer than that from either KNP or Chinese Meishan pigs. The difference between the Western pig breeds (BB genotype) and KNP or Meishan pigs (AA genotype) was due to an inserted 276-bp element and the 5-bp ACTTG.

**Conclusions:**

The polymorphism in *OGT* identified in this study may be used as an additional marker for determining the breed of origin among Meishan and the Western pig breeds. The length polymorphism suggests that the locus near *OGT* is not fixed in KNP. This marker would be relevant in determining the breed of origin in crossbred pigs between KNP pigs with known genotypes and the Western pig breeds with BB genotypes, thus confirming the contribution of the X chromosome from each breed.

## Background

The Korean native pig (KNP) is generally believed to have come from northern China to the Korean peninsula approximately 2000 years ago [1]. KNP pigs have black coat color in general and were at the brink of extinction in the 1980s due to their smaller animal size and slower growth rate [2]. Since then, effort has been made to restore the breed by bringing together the remaining stocks from a few provinces in the Republic of Korea [2-4]. As a result, the restored KNP has been registered as a breed in 2006 [3]. Genetic relationships between KNP, Chinese Meishan [4] and Western [4,5] pig breeds have been determined using microsatellite markers. To find additional breed-specific markers that are distinct among pig breeds, several genes including *O-linked N-acetylglucosamine transferase* (*OGT*) were investigated. *OGT* is located on chromosome X and catalyzes the post-translational addition of a single O-linked-β-N-acetylglucosamine (O-GlcNAc) on the hydroxyl groups of Ser and/or Thr residues of target proteins [6]. GlcNAcylation regulates cellular signaling and transcription processes in response to nutrients and stress, and has extensive crosstalk with phosphorylation [6]. OGT is also involved in nutrient sensing [7]. *OGT* has been mapped within the quantitative trait locus (QTL) affecting backfat depth, boar plasma FSH, and testicular weight using a length polymorphism within the intron 20 of *OGT* between Chinese and the Western pig breeds [8]. This genomic region located on chromosome X was investigated using Meishan (MS) x White composite (WC) crossbred boars [9]. Further, boars with MS alleles at the region had smaller testicles and lower total daily sperm production than boars with WC alleles [10]. In MS x WC crossbred boars, the length polymorphism in *OGT* was applicable to genotyping and identifying the breed of origin [8]. Therefore, the role of *OGT* and differential contribution of the X chromosome from each breed in crossbred pigs is worthy of further investigation. The first objective of this study was to determine whether the length polymorphism in the intron 20 of *OGT* was present in KNP and this polymorphism could be used as a breed-specific marker among KNP, Chinese Meishan, and the Western pig breeds. In addition, KNP pigs have been used to produce specialty meat and they have been crossbred to Landrace or Yorkshire breeds for growth and meat quality trait studies in Korea [11-13]. Thus, the second objective of this study was to determine whether the length polymorphism can be used as a breed-specific marker in screening the breed of origin in crossbred pigs between KNP with known genotypes and the Western pig breeds.

## Methods

### Experimental animals

The experimental protocol and standard operating procedures on experimental animals were reviewed and approved by the Institutional Animal Care and Use Committee of the National Institute of Animal Science, RDA (Suwon, Republic of Korea), in compliance with standard international regulations. Initially, 10 animals for each breed from the breeding stocks at the National Institute of Animal Science, Rural Development Administration, Republic of Korea were analyzed for the presence of the length polymorphism. The animals included 10 Duroc sows, 5 Landrace boars and 5 sows, 3 Yorkshire boars and 7 sows, 5 KNP boars and 5 sows, 1 Meishan boar, 8 sows and 1 unknown sex, and 10 Duroc x KNP crossbred sows. Subsequently, the number of pigs was increased to include 40 Duroc, 36 Landrace, 36 Yorkshire, 39 KNP, 10 Meishan, and 15 Duroc x KNP crossbred pigs (n = 176). Blood samples were collected and DNA was isolated.

### Primer design, PCR amplification, genotyping, and sequencing

Primers were designed to amplify across the intron 20 based on the porcine *OGT* cDNA (GenBank accession no. DQ400859) and the gene sequence in the Genome Browser for pig (http://genome.ucsc.edu/). The forward (2968F) and reverse (3083R) primers correspond to bases 2968–2987 and 3083–3059 of the porcine *OGT* cDNA (GenBank accession no. DQ400859), respectively, and the expected size of the PCR amplicon across intron 20 was 631 bp. PCR reactions were carried out in a 20-μl volume containing 30 ng of genomic DNA, 2 mM MgCl_2_, 10 pmol of each primer (*OGT*-2968F: 5′-GCACACCACAGGGATGGATG-3′ and *OGT-* 3083R: 5′- GCTCAAGACAACCTAAACAAGTAAG-3′), 200 μM dNTP, and 2 U Top-*Taq*™ DNA polymerase (Qiagen, Germany). Amplification was performed under the following PCR conditions: 10 min at 95°C; 35 cycles of 30 sec at 95°C, annealing for 30 sec at 60°C, and 1 min at 72°C; and a final extension of 5 min at 72°C. Length polymorphism among the pig breeds was determined after gel electrophoresis. Genotypes of the *OGT* gene among the Duroc, Landrace, Yorkshire, KNP, Meishan, and Duroc x KNP pigs (n = 176) were determined. A number of pigs were sequenced to confirm the *OGT* genotypes of Duroc, KNP, and Meishan pigs.

## Results and discussion

**Figure 1:**
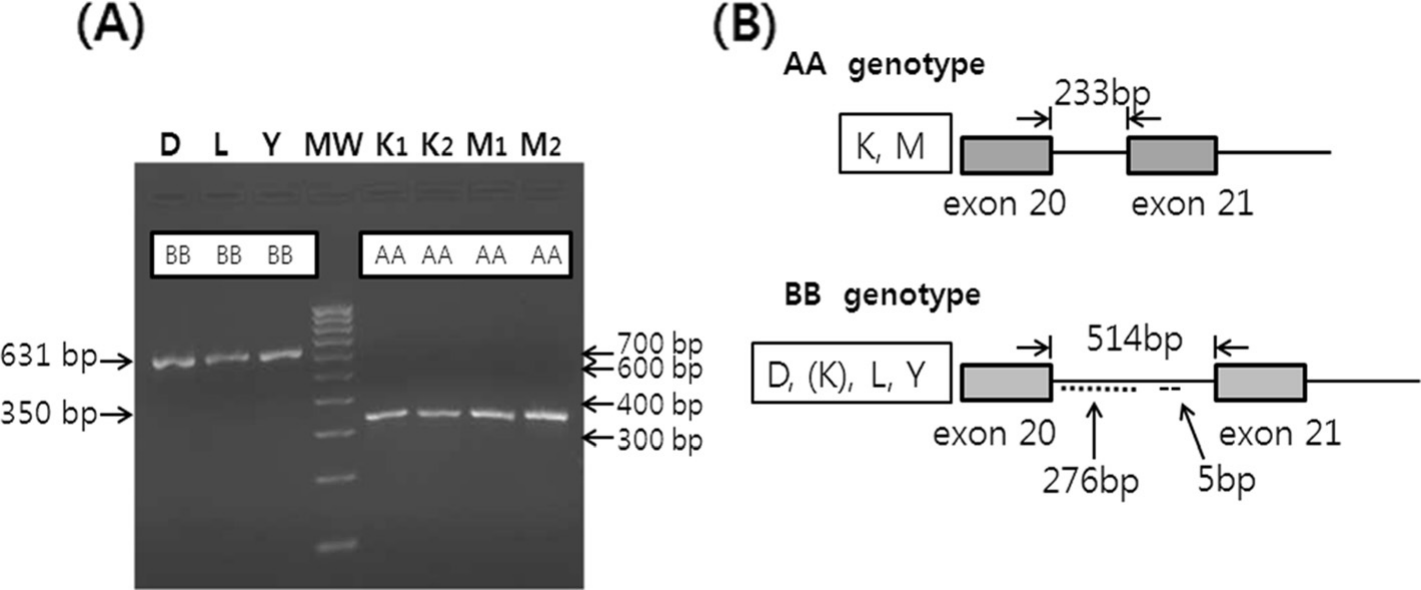
**Genotypes of** ***OGT*** **in different pig breeds and their schematic diagram.** **(A)**. The fragments containing the intron 20 of *OGT* from the Western pig breeds including Duroc (D), Landrace (L), and Yorkshire (Y) with BB genotypes (631 bp) were compared to those from Korean native pig (K) and Chinese Meishan (M) pigs with AA genotypes (350 bp). Single lane represents each breed for D, L, and Y (lanes 1–3) and two lanes are included for K (K1 and K2, lanes 5–6) and M (M1 and M2, lanes 7–8) with molecular weight (MW) marker. **(B)**. Schematic diagram comparing the intron 20 of *OGT* between K, M (above) with AA genotypes and the Western (D, [K], L, and Y) (below) pig breeds with BB genotypes. The intron 20 of *OGT* from K and M was 233 bp, whereas that from D, (K), L and Y was 514 bp. The longer intron 20 of *OGT* from the Western breeds contained an inserted 276-bp element (⋅⋅⋅⋅⋅) near the beginning of the intron and the 5 bp-ACTTG (---) insertion in the middle of the intron.

Length polymorphism in the intron 20 of *OGT* among the pig breeds was determined by PCR and gel electrophoresis (Figure 1A). Both strands of the genomic DNA corresponding to the intron 20 of *OGT* were amplified from KNP, Duroc, and Meishan boars, sequenced, and then submitted to the GenBank (accession no. JQ045376-7 and JQ579450). Amplification of a fragment containing the intron 20 of *OGT* by PCR revealed variations between KNP, Chinese Meishan and the Western pig breeds, including Duroc, Landrace, and Yorkshire. Fragments containing the intron 20 of *OGT* appeared to be the same among Duroc, Landrace, and Yorkshire pigs and were longer than those from either Meishan or KNP pigs (Figure 1A). In the initial study, using 10 purebred pigs from each breed, amplicons from all 10 pigs from each breed were the same size, except KNP pigs (data not shown). Amplicons from 8 out of 10 KNP pigs were the same, but those from the remaining two were different. The size of the fragment containing the intron 20 of *OGT* from Duroc boars was 631 bp (GenBank accession no. JQ045377), whereas those of Meishan (GenBank accession no. JQ579450) and 8 out of 10 KNP pigs were 350 bp (GenBank accession no. JQ045376). Sequencing indicated that the sizes of *OGT* intron 20 of the Duroc boars were 514 bp, while those from Meishan or KNP boars were 233 bp, resulting in a difference of 281 bp. The size and sequence of the intron 20 *OGT* from Duroc boars were identical to the one in the Genome Browser for pig (http://genome.ucsc.edu/). The 281-bp difference in the intron between Duroc and Meishan or KNP was due to an inserted 276-bp element near the beginning of the intron and the 5-bp ACTTG insertion in the middle of the intron (Figure 1B). These results are in agreement with a previous study on Meishan and White composite pigs. In that study, using 83 boars of 16 litters from inter se matings of 1/2 Meishan (MS) × 1/2 White composite (WC) parents, the breeds of origin of X chromosome QTL were confirmed based on this *OGT* polymorphism in the boars. The boars had either MS allele with AA genotype or WC allele with BB genotype (Kim JG, Nonneman D, Rohrer GA, unpublished observations).

When the entire intron 20 sequence of *OGT* from Duroc (GenBank accession no. JQ045376) was compared using the algorithm “Basic Local Alignment Search Tool (BLAST)”, it was revealed that several homologous regions to this sequence existed in the human chromosome X and in pig chromosomes. However, when the inserted 276-bp element of the intron 20 was compared using the BLAST, homologous regions existed only in several pig chromosomes, but not in any other species. The sizes of the *OGT* intron 20 from human, cattle, and mice were 240, 243, and 262 bp (http://genome.ucsc.edu/), respectively, and thus, they are similar to the 233-bp intron from either Meishan or KNP boars. Sequence homologies of the *OGT* intron 20 of Meishan and KNP boars, which do not contain the inserted 276-bp element and the 5-bp ACTTG of the *OGT* intron 20, with those of the human, cattle and mice were 76.3%, 69.5%, and 62.0%, respectively. This suggests that the 276-bp element and the 5-bp ACTTG of the *OGT* intron 20 in Duroc and other Western pig breeds may have been inserted differentially during the domestication process in different regions.

**Figure 2:**
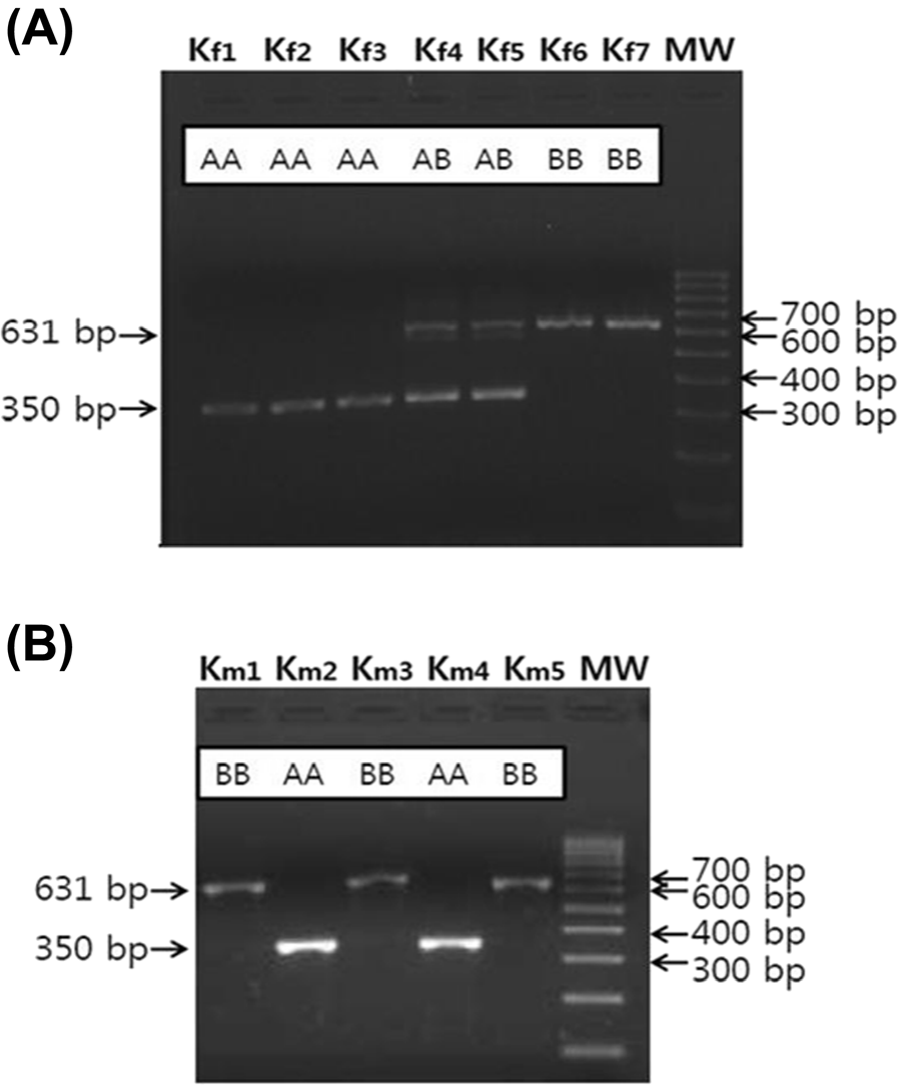
**Representative pictures showing the genotypes of** ***OGT*** **in KNP pigs. (A)**. Representative picture showing the genotypes of *OGT* in KNP sows. Fragments amplified by PCR containing the intron 20 of *OGT* from seven Korean native pig sows (from Kf1 to Kf7) with molecular weight (MW) marker are shown. The 350-bp fragment was amplified in three sows (Kf1, Kf2, and Kf3) with AA genotypes, and the 350-bp and 631-bp fragments were amplified in two sows (Kf4 and Kf5) with AB genotypes, and the 631-bp fragment was amplified in two sows (Kf6 and Kf7) with BB genotypes. **(B)**. Representative picture showing the genotypes of *OGT* in KNP boars. Fragments amplified by PCR containing the intron 20 of *OGT* from five Korean native pig boars (from Km1 to Km5) with molecular weight (MW) marker are shown. The 631-bp fragment was amplified in three boars (Km1, Km3, and Km5) with BB genotypes and the 350-bp fragment was amplified in two boars ‘(Km2 and Km4) with AA genotype.

**Figure 3:**
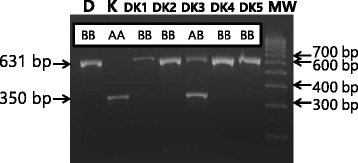
**Genotypes of** ***OGT*** **from D, K, and D x K crossbred sows.** After two generations of crossbreeding between Duroc (D) and Korean native pig (K), the genotypes of four out of five Duroc x Korean native pig crossbred (DK) sows (DK1, 2, 4, and 5 in lanes 3, 4, 6 and 7) were BB, having the same 631-bp fragment amplified as D. The genotype of one (DK3 in lane 5) of the five DK sows was AB, having both the 631 bp fragment, which is the same as the Duroc sow (in lane 1) and the 350-bp fragment, which is the same as the KNP sow (in lane 2).

There was a variation in the intron 20 *OGT* among the 10 KNP pigs in the initial study. Unexpectedly, 8 out of the 10 KNP pigs had a short intron, which is the same as the Meishan pigs, whereas 2 KNP pigs had a long intron, which is the same as the Western pigs (data not shown). When additional pigs were analyzed, KNP pigs had short and long intron 20 of *OGT*, and others were heterozygotes, and thus the genotypes were designated as AA, BB, and AB, respectively (Figure 2A, B). In contrast to the KNP pigs, the genotypes of *OGT* in Meishan pigs and the Western breeds were fixed as AA and BB, respectively (Table 1). When genotypes of 39 KNP pigs were analyzed, there were 16 AA (41.0%), 13 AB (33.3%), and 10 BB (25.6%). When we compared the genotypes of *OGT* between KNP pigs born in 2009 and in 2010 or 2011, the frequency of allele A decreased slightly in 2010 or 2011 in comparison to 2009. The genotypes of 18 pigs born in 2009 were 8 AA (44.4%), 6 AB (33.3%), and 4 BB (22.2%). However, the genotypes of 21 pigs born in 2010 or 2011 were 8 AA (38.1%), 7 AB (33.3%), and 6 BB (28.6%). The results suggest that the locus is not fixed near *OGT* in KNP pigs. The observed frequency reduction of genotype AA among KNP pigs born in 2011 or 2012 could be due to the sampling bias of including more sows born from parents of AB or BB genotypes (Table 1). However, we cannot rule out that the reduction is due to the heterogametic state of boars or the result of genetic drift in males. After two generations of crossbreeding between Duroc with KNP pigs, all five Duroc x KNP (DK) crossbred boars and eight of 10 DK crossbred sows had BB genotypes, which is the same as Duroc boars, while the remaining two DK crossbred sows had AB genotypes (Table 1) (Figure 3).

**Table 1:** **Frequency of** ***OGT*** **genotypes among different pig breeds**

	**AA**	**AB**	**BB**	**Total**
Duroc	0	0	40	40
Landrace	0	0	36	36
Yorkshire	0	0	36	36
KNP	16	13	10	39
(KNP boar)^a^	(7)	(0)	(3)	(10)
(KNP sow)^b^	(9)	(13)	(7)	(29)
Meishan	10	0	0	10
Duroc x KNP	0	2	13	15
Total	28	13	135	176

^a^^,^^b^Boars and sows of KNP pigs were calculated within ( ).

## Conclusions

In conclusion, the length polymorphism in the intron 20 of *OGT* may be used as an additional marker for determining the breed of origin among Chinese Meishan and the Western pig breeds including Duroc, Landrace, and Yorkshire. The polymorphism identified in this study suggests that the locus near *OGT* is not fixed in KNP pigs, and this marker may supplement the restoration effort of KNP as an additional mean to verify the origin of the breed near this locus. It would be also relevant in determining the breed of origin in crossbred pigs between KNP with known genotypes and Duroc or other Western breeds with BB genotypes, and thus confirming the contribution of the X chromosome from each breed.
